# Anatomical location matters: risk modelling and surgical outcomes in central lymph node metastasis of isthmus PTC

**DOI:** 10.3389/fendo.2026.1922413

**Published:** 2026-09-28

**Authors:** Yuexin Yu, Wenqing Cai, Guang Zhang, Qingfeng Fu, Yishen Zhao, Jingting Li, Tie Wang, Jie Liu, Kangping Li, Zhuo Chen, Carla Colombo, Gianlorenzo Dionigi, Xiaoli Liu

**Affiliations:** 1 Department of Thyroid Surgery, China–Japan Union Hospital of Jilin University, Changchun, China; 2 Department of Burn and Plastic Surgery, Jinan City People's Hospital Affiliated to Shandong First Medical University, Jinan, Shandong, China; 3 Department of Thyroid and Breast Surgery, Weifang Hospital of Traditional Chinese Medicine, Weifang, Shandong, China; 4 Department of Pathophysiology and Transplantation, University of Milan, Milan, Italy; 5 Endocrine Oncology Unit, Istituto Auxologico Italiano IRCCS, Milan, Italy; 6 Division of General Surgery, Endocrine Surgery Section, Istituto Auxologico Italiano IRCCS, Milan, Italy

**Keywords:** cervical lymph node metastasis, isthmus, neck dissection, nomograms, papillary thyroid carcinoma, prognosis, retrospective studies, risk factors

## Abstract

**Background:**

Isthmus papillary thyroid carcinoma (iPTC) represents a distinct clinical entity with a higher propensity for central lymph node metastasis (CLNM) and greater local aggressiveness compared to lobe-confined PTC. Despite increasing recognition of its unique risk profile, controversy persists regarding optimal surgical management, particularly for small or indolent tumours.

**Methods:**

This retrospective cohort study included 319 patients with histologically confirmed iPTC who underwent comprehensive ultrasonographic assessment and thyroid surgery from February 2019 to June 2024 at a high-volume tertiary centre. Lobe-confined PTC cases were matched as controls using propensity score methodology. Clinicopathological features, risk factors for CLNM, surgical outcomes, and recurrence patterns were analysed using univariate and multivariate analyses and ROC/AUC modelling. A predictive nomogram for CLNM was developed and internally validated using 1,000 bootstrap resamples with optimism correction.

**Results:**

The iPTC cohort exhibited a significantly higher CLNM burden than matched lobe-confined PTC controls, independent of tumour size or multiplicity. Multivariate analysis identified male sex, increased isthmus tumour diameter, and calcification as independent preoperative predictors of CLNM. The derived nomogram demonstrated acceptable discrimination (optimism-corrected AUC 0.722, 95% CI 0.645–0.802) and internal calibration. Surgical extent (total thyroidectomy versus less extensive resection) was associated with increased perioperative morbidity but did not improve early recurrence rates in selected cases. There were no lymph node or distant recurrences on intermediate-term follow-up.

**Conclusions:**

Isthmus location independently increases central nodal metastatic risk in PTC. Risk modelling with simple clinical and ultrasonographic factors quantifies the probability of central lymph node metastasis and highlights the trade-off between more extensive thyroid resection and increased perioperative morbidity. We did not observe an early recurrence or survival advantage associated with more extensive surgery in this cohort, within the limits of intermediate follow-up and routine central dissection; instead, they support individualised, risk-adapted decision-making that requires validation in prospective outcome studies.

## Introduction

Papillary thyroid carcinoma (PTC) is the predominant histotype amongst thyroid malignancies, accounting for up to 85% of cases (1). Whilst the thyroid lobes are the most frequent site, a clinically relevant subset of PTCs—estimated at 1.0% to 9.2%—arise in the isthmus (2, 3). Isthmic PTC (iPTC) is associated with a higher incidence of extrathyroidal extension, multifocality, and central lymph node metastasis due to its unique anatomical and lymphatic context (4). Despite these aggressive features and a well-documented risk profile, guidance for optimal management, especially for small (≤1 cm) or low-risk iPTC, remains limited and controversial.

In addition, it is well established that occult central lymph node metastases are common in papillary thyroid carcinoma, including iPTC. However, several studies have not shown that routine prophylactic central neck dissection in otherwise low-risk disease improves disease-specific survival or durable recurrence outcomes. In this context, the clinical question is not simply whether occult metastases exist, but how best to quantify their probability and balance any potential staging benefit against increased surgical morbidity. The present study therefore focuses on (1) characterising patterns and predictors of central lymph node metastasis in iPTC, distinguishing anatomical risk from clinical presentation, and (2) evaluating how surgical extent affects perioperative outcomes, without assuming that removal of occult nodes itself improves long-term survival.

This study uses a comprehensive, stratified approach—distinguishing patients by lesion location, CLNM status, and surgical technique—to analyse the patterns and determinants of metastatic risk. The multifactorial analysis strengthens the evidence base for tailoring surgical strategy and risk stratification in iPTC, providing nuanced, clinically actionable data to inform contemporary practice and guideline development.

## Materials and methods

### Study design and setting

This retrospective observational cohort study was conducted at a high-volume tertiary endocrine surgery centre and adhered to established reporting standards for observational research in surgical oncology. Clinical, radiological, and outcome data were systematically collected for 319 consecutive patients with histologically confirmed isthmus papillary thyroid carcinoma (iPTC) who underwent comprehensive preoperative ultrasonography and surgical intervention from February 2019 to June 2024. To minimise confounding and selection bias, contemporaneously managed patients with lobe-confined PTC were used as comparators, matched to the iPTC cohort using propensity score matching (PSM) methodology, incorporating key covariates such as age, sex, tumour diameter, and multifocality.

### Ethics approval and study protocol

This study was conducted in full compliance with the principles of the Declaration of Helsinki and relevant national and institutional research ethics guidelines, including those of the Chinese Medical Association and the American Thyroid Association. The research methodology was approved by the Ethics Committee of Jilin University China–Japan Friendship Hospital, with approval number (2023070307). The study protocol, including the use of patient data and follow-up, was formally reviewed and approved by the Institutional Review Board (IRB) of the participating centre prior to commencement. All patients provided written informed consent for the use of their anonymised clinical, imaging, and laboratory data for research purposes. Rigorous adherence to patient privacy and data security standards was maintained throughout data collection, handling, and analysis, in accordance with best-practice recommendations for clinical thyroid research. The manuscript was prepared according to the STROBE guidelines for observational studies.

### Inclusion and exclusion criteria

Inclusion criteria were: (1) patients who underwent primary thyroidectomy at the study institution, with postoperative histopathological confirmation of PTC; (2) availability of complete and sequential preoperative, intraoperative, and postoperative clinical documentation; (3) no prior thyroid or neck surgery, ensuring an unselected, treatment-naïve cohort for uniform baseline assessment.

Exclusion criteria were: (1) incomplete or missing clinical records, or prior ablative treatment (e.g. radiofrequency or ethanol ablation) performed either at the study centre or elsewhere; (2) presence of distant metastases or other concomitant malignancies as detected by preoperative imaging, to eliminate confounding from advanced or multifocal disease; (3) history of previous neck surgery, which may affect anatomical dissection planes, lymphatic mapping, and outcome assessment.

### Preoperative assessment

All patients underwent high-resolution colour Doppler ultrasonography of the thyroid and cervical lymph nodes, performed by experienced radiologists using standardised protocols consistent with American Thyroid Association and international guidelines (1). Ultrasound assessment included precise documentation of lesion location, maximum diameter, aspect ratio (taller-than-wide or wider-than-tall), margins (circumscribed or not), internal echogenicity (isoechoic, hypoechoic, or markedly hypoechoic), presence and type of calcification (microcalcifications or macrocalcifications), extrathyroidal extension, and central compartment lymph node status (1, 5). A tumour was strictly classified as isthmus PTC (iPTC) if the geometric centre—determined by the intersection of the lesion’s long and short axes—lay within the thyroid isthmus, defined as the region between two parallel vertical lines tangent to the lateral borders of the trachea on transverse ultrasound imaging (5). Preoperative laryngoscopy was performed to assess vocal fold mobility, and laboratory evaluations included thyroid function, calcium, parathyroid hormone, calcitonin, TPOAb, and TgAb, allowing comprehensive endocrine and oncological risk stratification (1).

### Patient stratification and surgical management scheme

Patients were stratified into clinically meaningful subgroups to delineate oncological risk and guide surgical management. Grouping parameters included: (1) anatomical location—distinguishing those with primary or exclusive isthmus involvement (iPTC group) from those with disease confined to the thyroid lobe (PTC group), according to consensus criteria; (2) central lymph node metastasis (CLNM) status—where iPTC patients were subclassified as CLNM-positive or CLNM-negative based on comprehensive ultrasonographic and intraoperative pathological assessment; (3) operative approach for simple iPTC—defining Group A (isthmusectomy alone), Group B (isthmusectomy with unilateral lobectomy or subtotal bilateral lobectomy), and Group C (total thyroidectomy), reflecting current guideline recommendations for the extent of resection in isthmus-located tumours.

For the purposes of this analysis, central lymph node status was further subclassified as clinically evident versus occult metastasis. Clinically evident CLNM was defined as radiologically or clinically suspicious nodes (abnormal ultrasound features and/or intraoperative macroscopic suspicion) confirmed on final histopathology. Occult CLNM was defined as metastatic involvement of central nodes that were not suspected pre- or intra-operatively (cN0) and were identified only on routine pathological examination of prophylactically dissected specimens. Where possible, patients were categorised as cN0/pN0, cN0/pN+, or cN1/pN+.

All surgical subsets underwent standardised central compartment lymph node dissection in accordance with American Thyroid Association and international best-practice protocols, enabling robust comparison of surgical outcomes across anatomical and technical strata.

The surgical management scheme for simple isthmus papillary thyroid carcinoma is illustrated in **Figure 1**.

**Figure 1:**
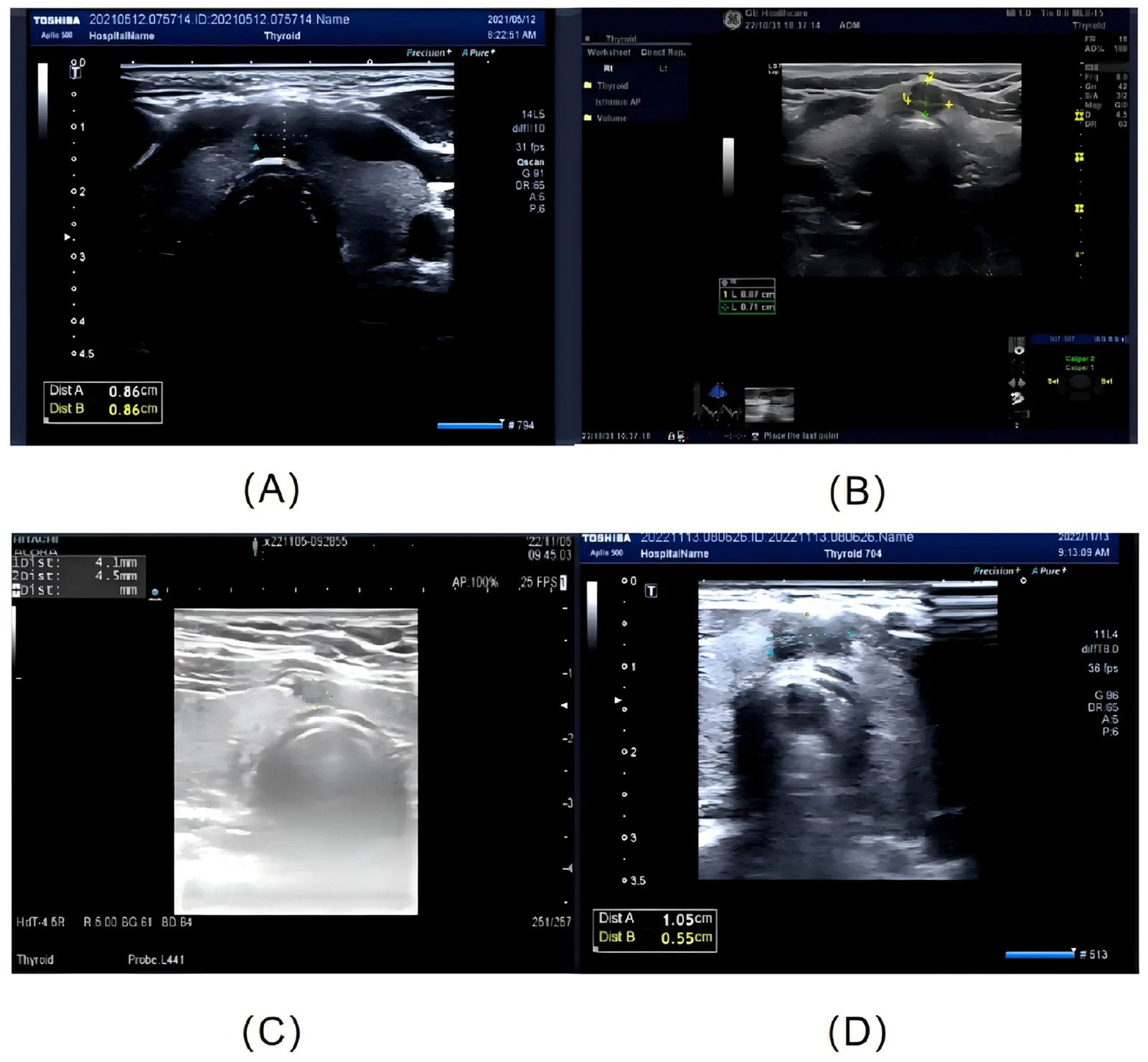
Surgical management scheme for simple isthmus papillary thyroid carcinoma. **(A)** Preoperative ultrasound showing isthmus PTC; **(B)** Intraoperative view of isthmus resection; **(C)** Specimen photography; **(D)** Histopathological confirmation.

### Surgery

All surgical procedures were performed by two senior endocrine surgeons, each with over ten years of specialised experience, and were carried out in strict accordance with the latest Chinese (2nd Edition) and 2025 American Thyroid Association management guidelines for differentiated thyroid cancer (1, 7). Under general anaesthesia, patients were positioned supine with neck extension facilitated by a shoulder roll to optimise surgical exposure. Intraoperative nerve monitoring (IONM) was systematically employed for continuous assessment of recurrent laryngeal nerve (RLN) integrity (6).

A low-arched transverse cervical incision was made to maximise cosmetic and anatomical outcomes, followed by meticulous, stepwise dissection through the skin, subcutaneous tissue, and platysma. Nanochar^®^ or a comparable carbon nanoparticle suspension was used for intraoperative parathyroid gland mapping and protection, as recommended in current guideline updates (7, 8). The thyroid isthmus was fully mobilised, transected, and extracapsular dissection of glandular tissue was performed, ensuring anatomical preservation. Standardised central compartment (level VI) lymph node dissection was conducted for all indicated cases, with rigorous documentation of RLN function, parathyroid management, and intraoperative findings, in line with contemporary evidence-based protocols for the surgical treatment of thyroid malignancy (1, 7).

### Quantitative imaging analysis and tumour measurement

Preoperative and intraoperative tumour measurements were systematically acquired and analysed using the open-source ImageJ software platform (National Institutes of Health, Bethesda, MD, USA), specifically utilising the FIJI (Fiji Is Just ImageJ) distribution and validated plugins for quantitative imaging (9). On digital ultrasound and photographic images, trained clinicians delineated regions of interest (ROIs) corresponding to tumour boundaries. Tumour size (maximum diameter), area, and perimeter were computed automatically, with standardised calibration for pixel-to-millimetre conversion, ensuring objective and reproducible quantification across modalities.

ImageJ/FIJI-based morphometric analysis is well validated for cancer image analysis, offering advantages over manual methods in speed, accuracy, and reduced observer bias. Specific FIJI plugins, such as CollagenFitJ and other tumour-mapping macros, further enable detailed assessment of tumour and peritumoural tissue architecture, and have demonstrated technical and clinical validity in thyroid, breast, and other oncological contexts (10). All data underwent intra- and interobserver reliability checks to confirm methodological rigour and analytical consistency.

### Comprehensive clinical data collection and quality assurance

Clinical, imaging, and laboratory data were rigorously collected using standardised protocols to ensure methodological consistency and analytical depth. Baseline demographic variables included age, sex, and BMI (with obesity defined as BMI >25 kg/m², consistent with WHO Asian criteria). Preoperative laboratory analyses included thyroid peroxidase antibody (TPOAb), thyroglobulin antibody (TgAb), and corrected serum calcium, all of which have established relevance for operative risk and endocrine assessment.

Ultrasound parameters were systematically documented: tumour location (isthmus/lobe), maximum orthogonal diameter, interface margins (smooth, microlobulated, or irregular), aspect ratio (height/width), echogenicity, and the presence or character of calcifications, in line with ATA TIRADS and international risk stratification systems (1). Surgical case report forms captured operative approach, operative time, technique (open, endoscopic, robotic), central lymph node (CLN) yield, completeness of dissection, histopathology (including CLN metastasis, capsular and extrathyroidal invasion, TNM stage), as well as perioperative and postoperative laboratory outcomes (serum calcium, tube drainage volume, and duration of tube placement).

Hashimoto’s thyroiditis was defined by positivity for either TPOAb or TgAb, whilst postoperative hypoparathyroidism was documented for corrected serum calcium <2.2 mmol/L (1). All data were prospectively entered and retrospectively validated according to best-practice oncological quality standards.

### Postoperative surveillance and risk-adapted follow-up

All patients were managed postoperatively in accordance with international thyroid cancer surveillance guidelines. Thyroid-stimulating hormone (TSH) suppression therapy was initiated for all patients following resection, tailored to risk stratification and current consensus recommendations. Scheduled follow-up included comprehensive thyroid function tests and high-resolution neck ultrasonography at 3–6 month intervals for dynamic monitoring of residual or recurrent disease. When suspicion for recurrence or regional metastasis increased—indicated by rising serum thyroglobulin, abnormal imaging, or clinical symptoms—second-level imaging (neck and/or chest CT) was performed, and suspicious cervical lesions underwent ultrasound-guided fine-needle aspiration biopsy, in line with ATA standards.

Recurrence-free survival (RFS) was defined as the interval from initial surgery to the date of first confirmed disease recurrence, metastatic progression, or last negative follow-up (censored 10 November 2024). This systematic, risk-adapted surveillance protocol reflects contemporary best practice in differentiated thyroid cancer follow-up, optimising early detection and individualised management.

### Endpoints

Primary endpoints: (1) Comprehensive characterisation of the prevalence, anatomical patterns, and independent risk factors associated with cervical lymph node metastasis (CLNM) in isthmus papillary thyroid carcinoma (iPTC); (2) Development and internal validation of a multivariable prediction nomogram for CLNM risk.

Secondary endpoints: (1) Evaluation of individualised, risk-adapted surgical strategies; (2) Comparative effectiveness analysis of surgical techniques; (3) Correlative analysis of adverse histopathological features (capsular invasion, extrathyroidal extension) and their impact on clinical course and recurrence; (4) Documentation and evaluation of intraoperative technical findings, conversion rates, and indications for escalation of care; (5) Incidence and risk factors for postoperative complications, specifically hypocalcaemia and recurrent laryngeal nerve injury, to benchmark perioperative safety.

### Statistical analysis

Statistical analysis was conducted using rigorous, contemporary methodology tailored to observational surgical oncology research. Propensity scores for isthmus versus lobe-confined tumour location were estimated using logistic regression including sex, age, maximum tumour diameter, and multifocality, selected *a priori* as established baseline confounders related to tumour burden and nodal metastatic risk. Patients were matched 1:1 by nearest-neighbour matching without replacement, using a caliper of 0.02, yielding 268 closely matched pairs. Covariate balance before and after matching was assessed using standardised mean differences (SMDs); an absolute SMD < 0.10 was considered evidence of acceptable balance. Sonographic characteristics and nodal status were not incorporated into the primary matching model because they were evaluated as location-associated clinical features and may lie on the pathway linking tumour location with nodal involvement.

For quantitative comparisons, continuous variables were assessed using Student’s t-test (or Mann–Whitney U test for nonparametric distributions), whilst categorical variables were analysed using the chi-square test or Fisher’s exact test, as appropriate. Receiver operating characteristic (ROC) curve analysis was used to identify optimal cutoff values for tumour size in predicting central lymph node metastasis (CLNM), with area under the curve (AUC) as the discrimination metric. Multivariate logistic regression modelling was used for risk factor analysis, and weighted regression coefficients enabled the construction of an individualised prediction nomogram and composite risk score, stratifying patients into low-, intermediate-, or high-risk categories for nodal disease.

Internal validation was performed using 1,000 bootstrap resamples. For each resample, the model was refitted and evaluated in both the bootstrap sample and the original dataset. Optimism-corrected estimates of model discrimination and calibration were derived from the average optimism across resamples.

All statistical calculations were performed using IBM SPSS Statistics version 29.0 (IBM Corp., Armonk, NY, USA). Statistical significance was determined at a two-sided P < 0.05. Analytical rigour was ensured by systematic model validation and adherence to contemporary recommendations for statistical reporting in thyroid cancer outcomes research.

### Predictive model and nomogram

The number of central lymph nodes removed was considered an operative/pathological variable and was excluded from the final preoperative prediction model because nodal yield may influence the probability of detecting occult metastasis. The nomogram was restricted to variables available before surgery: sex, tumour diameter, and suspicious calcification.

The overall study flow chart and group allocation are shown in **Figure 2**.

**Figure 2:**
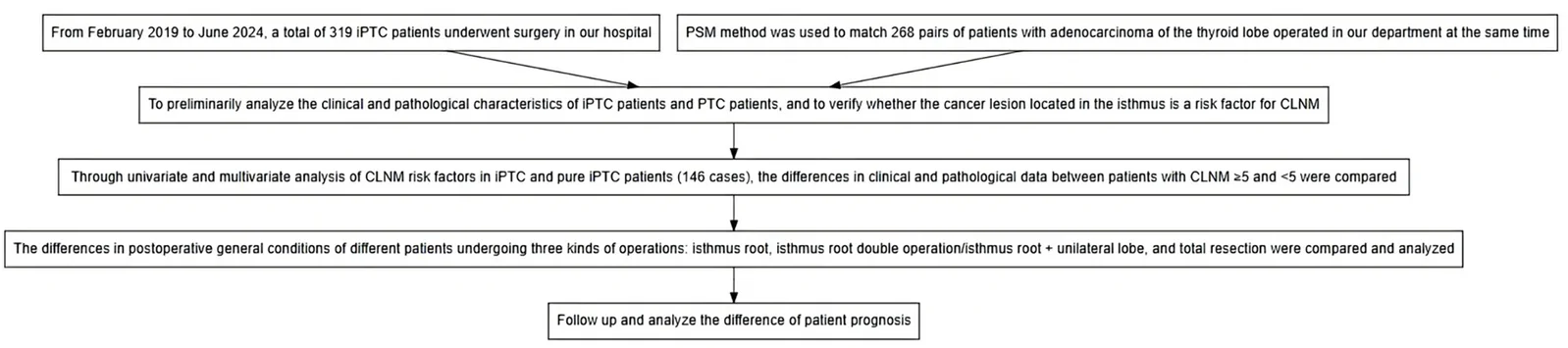
Flowchart of the study design, inclusion/exclusion criteria, and patient allocation for the iPTC and matched lobe-confined PTC cohorts.

## Results

### Baseline clinicopathological and ultrasonographic profile of the iPTC cohort

A total of 319 patients with histologically confirmed isthmus papillary thyroid carcinoma (iPTC) were included. The clinical and pathological features of 319 iPTC patients are presented in **Table 1**. The cohort comprised 257 females (80.6%) and 62 males (19.4%), with a mean age of 43.9 ± 10.5 years (range 19–71); 48.3% (n = 154) were aged ≥45 years, and 16.6% (n = 53) were ≥55 years. The median body mass index (BMI) was 24.7 (IQR 22.9–27.8), with 61.8% (n = 197) meeting the criteria for overweight status. Coexistent chronic lymphocytic thyroiditis was pathologically established in 81 patients (25.4%). High-resolution ultrasonography revealed that most iPTC nodules were subcentimetre, with a median maximal diameter of 0.61 cm (range 0.45–0.89 cm). Micro- or macrocalcifications were identified in 52% (n = 166), an aspect ratio >1 (taller-than-wide) in 53.9% (n = 172), and irregular or ill-defined margins in 84.6% (n = 270). Postoperative pathological assessment showed that 48.6% (n = 155) had central lymph node metastases (CLNM), of whom 8.8% (n = 28) had ≥5 metastatic central lymph nodes, and 17.6% (n = 56) demonstrated histological evidence of thyroid capsule invasion, reflecting the aggressive clinicopathological profile often encountered in isthmus-located PTC.

**Table 1:** Clinicopathological characteristics of 319 iPTC patients.

Clinicopathological features	Number (%) n=319	Mean ± SD/median (IQR)
Gender		
Male	62 (19.4)	
Female	257 (80.6)	
Age (years)		43.9 ± 10.5
≥45	154 (48.3)	
≥55	53 (16.6)	
BMI (kg/m²)		24.7 (22.9, 27.8)
<25	122 (38.2)	
≥25	197 (61.8)	
Hashimoto’s thyroiditis		
Yes	81 (25.4)	
No	238 (74.6)	
Isthmus tumour diameter (cm)		0.61 (0.45, 0.89)
<1	259 (81.2)	
≥1	60 (18.8)	
Calcification		
Yes	166 (52.0)	
No	153 (48.0)	
Aspect ratio >1		
Yes	172 (53.9)	
No	147 (46.1)	
Unclear borders		
Yes	270 (84.6)	
No	49 (15.4)	
CLNM		
Yes	155 (48.6)	
No	164 (51.4)	
CLNM ≥5		
Yes	28 (8.8)	
No	291 (91.2)	
Invasion of the thyroid capsule		
Yes	56 (17.6)	
No	263 (82.4)	
TNM stage		
I	302 (94.7)	
II	17 (5.3)	

### Clinicopathological profile in simple isthmic papillary thyroid carcinoma

A total of 142 patients with simple iPTC, defined as tumours strictly confined to the isthmus, were analysed for detailed clinical, ultrasonographic, surgical, and pathological parameters. The detailed clinicopathological features of these 142 patients are provided in **Supplementary Table S1**.

### Comparative clinicopathological analysis: matched iPTC versus lobar PTC cohorts

Using 1:1 nearest neighbour propensity score matching (gender, age, tumour diameter, multifocality, caliper 0.02), 268 balanced pairs of isthmus-origin (iPTC) and lobe-only PTC were identified, eliminating baseline demographic and multiplicity differences. After adjusting for gender, age, tumour diameter, and number of cancer foci in two patient groups, the clinical-pathological characteristics after PSM are shown in **Table 2**. Median tumour size was nearly identical (iPTC: 0.60 cm [IQR: 0.45–0.86], PTC: 0.60 cm [IQR: 0.47–0.89]; P = 0.561). However, iPTC exhibited a distinctly more aggressive nodal profile: the number of central lymph node metastases (CLNMs) was higher (P = 0.016), as were the incidence of nodal metastasis (49.6% vs 39.9%, P = 0.024), the CLNM ratio (P = 0.039), and the proportion of patients with ≥5 CLNMs (7.5% vs 3.4%, P = 0.036). There were no statistically significant differences between groups for ultrasonic characteristics (calcifications, aspect ratio >1, or margin regularity), peritumoural invasion, or TNM staging. These findings demonstrate a persistently elevated lymph node metastatic burden in iPTC, even after rigorous covariate adjustment, supporting the assertion that isthmus location independently confers nodal aggressiveness irrespective of tumour size or multiplicity.

**Table 2:** Comparative analysis of clinical and pathological features between propensity score matched iPTC and lobe-confined PTC patients (n = 268 matched pairs).

Clinicopathological features	iPTC (n=268)	PTC (n=268)	Statistic	P-value
Gender			0.000	1.000
Male	53	53		
Female	215	215		
Age (years)	43.45 ± 10.53	42.99 ± 9.56	-0.532	0.595
≥45	225	236		
≥55	43	32	1.876	0.171
Isthmus tumour diameter (cm)	0.60 (0.45, 0.86)	0.60 (0.47, 0.89)	-0.581	0.561
<1	221	216		
≥1	47	52	0.310	0.578
Calcification			0.000	1.000
Yes	142	142		
No	126	126		
Aspect ratio >1			3.777	0.052
Yes	151	173		
No	117	95		
Unclear borders			1.328	0.240
Yes	230	239		
No	38	29		
CLN number removed	6 (3, 9)	5 (3, 8)	-1.386	0.166
CLNM number	0 (0, 2)	0 (0, 1)	-2.441	0.016
CLNM ratio (%)	0 (0, 33.3)	0 (0, 28.2)	2.065	0.039
CLNM			5.100	0.024
Yes	133	107		
No	135	161		
CLNM ≥5			4.411	0.036
Yes	20	9		
No	248	259		
Invasion of the thyroid capsule			0.136	0.712
Yes	40	37		
No	228	231		
TNM stage			0.351	0.554
I	253	256		
II	15	12		

### Univariate risk factor analysis for central lymph node metastasis in iPTC

Of 319 patients with isthmus papillary thyroid carcinoma, 155 (48.6%) were classified as CLNM-positive on postoperative pathology. Comparative univariate analysis showed significant associations with male sex (P = 0.002) and younger age (mean 42.08 ± 10.75 vs 45.57 ± 10.00 years, P = 0.003; both patient age <45 and <55 years were underrepresented in the CLNM-positive subgroup, P = 0.008 and P = 0.042, respectively). Median BMI and rates of overweight did not differ by nodal status.

As detailed in **Table 3**, CLNM-positive patients had larger isthmus tumour diameters (P < 0.001) and a higher frequency of suspicious calcifications on ultrasound (61.3% vs 43.3%, P = 0.001). Other sonographic features—aspect ratio, margin irregularity—as well as multifocality and perineural invasion, showed no statistically significant differences. Operatively, CLNM-positive patients underwent more extensive central lymph node dissections (P < 0.001) and were less likely to be assigned TNM stage I (P < 0.001). These results highlight that male sex, younger age, increased tumour diameter, and the presence of calcification are important univariate risk indicators for central nodal metastasis in iPTC, consistent with published multi-cohort analyses implicating younger age, larger tumour size, and aggressive sonopathological features as key risk factors in papillary thyroid carcinoma.

**Table 3:** Univariate analysis of clinical and pathological features in iPTC CLNM (n = 319).

Clinicopathological features	CLNM+ (n=155)	CLNM- (n=164)	Statistic	P-value
Gender			9.478	0.002
Male	41	21		
Female	114	143		
Age (years)	42.08 ± 10.75	45.57 ± 10.00	2.997	0.003
<45	92	73	7.030	0.008
≥45	63	91		
<55	136	130	4.130	0.042
≥55	19	34		
BMI (kg/m²)	25.1 (23.0, 28.1)	24.4 (22.9, 27.3)	-0.876	0.381
<25	56	66	0.571	0.450
≥25	99	98		
Hashimoto’s thyroiditis			0.122	0.727
Yes	38	43		
No	117	121		
Tumour diameter (cm)	0.67 (0.51, 1.04)	0.56 (0.40, 0.74)	-4.396	<0.001
Ultrasound multifocal			0.050	0.822
Yes	87	90		
No	68	74		
Calcification			10.342	0.001
Yes	95	71		
No	60	93		
Aspect ratio >1			2.897	0.089
Yes	76	96		
No	79	68		
Unclear borders			0.316	0.574
Yes	133	137		
No	22	27		
Number of CLN removed	6 (5, 11)	5 (2, 8)	-4.910	<0.001
Invasion of the thyroid capsule			2.907	0.088
Yes	33	23		
No	122	141		
TNM stage			14.901	<0.001
I	139	163		
II	16	1		

### Independent predictors of CLNM: multivariate logistic regression and ROC curve analysis

All variables with univariate significance (P < 0.1) were included in a multivariate logistic regression model to identify predictors of central lymph node metastasis (CLNM) amongst iPTC patients. After excluding the number of central lymph nodes removed as a procedure-dependent variable, male sex (P = 0.004), increased isthmus tumour diameter (P = 0.001), and suspicious calcification (P = 0.021) remained statistically significant independent preoperative risk factors for CLNM (**Table 4**). Age was also retained in the model as a significant protective factor (OR 0.97, 95% CI 0.95–0.99, P = 0.005). Receiver operating characteristic (ROC) curve analysis confirmed that isthmus tumour diameter was a significant single-variable predictor of CLNM, with an area under the curve (AUC) of 0.642 (95% CI: 0.582–0.702, P < 0.001). The optimal diameter cutoff for discriminating risk—maximising sensitivity (81.3%) and specificity (39.6%)—was 0.475 cm. These findings reinforce the dominant role of tumour size and sex in CLNM risk stratification for iPTC, supporting the development of individualised risk-based management algorithms. .

**Table 4:** Multifactorial logistic regression analysis of clinicopathological characteristics predicting CLNM in iPTC (n = 319) — Revised model (preoperative variables only).

Clinicopathological features	B	OR	95% CI	P-value
Gender (Male)	0.887	2.43	1.32 – 4.47	0.004
Age (per year)	-0.033	0.97	0.95 – 0.99	0.005
Tumour diameter (cm)	1.035	2.81	1.52 – 5.19	0.001
Suspicious calcification	0.566	1.76	1.09 – 2.85	0.021

The ROC curve for isthmus tumour diameter predicting CLNM is presented in **Figure 3**.

**Figure 3:**
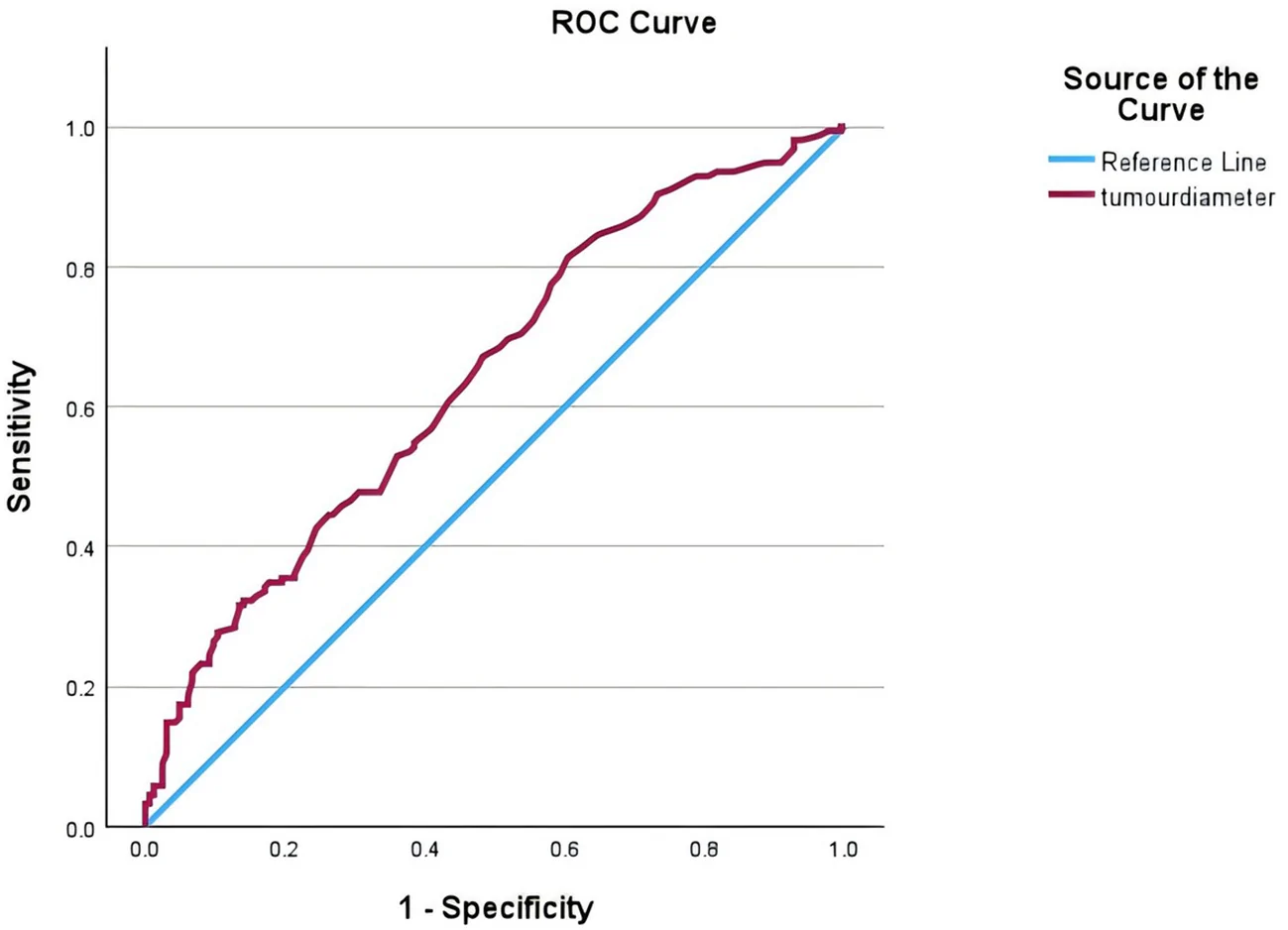
ROC curve of iPTC isthmus tumour diameter predicting CLNM (n = 319). AUC = 0.642 (95% CI: 0.582–0.702, P < 0.001). Optimal cutoff = 0.475 cm (sensitivity 81.3%, specificity 39.6%).

### Clinical versus occult central lymph node metastasis

Amongst patients with available cN-status documentation, most central lymph node metastases were occult, occurring in patients staged as cN0 but found to be pN+ on final pathology, whilst a smaller subset presented with clinically or sonographically evident nodal disease pre- or intraoperatively. In both iPTC and propensity-matched lobe-confined PTC, the prevalence of occult CLNM was high, consistent with previous multicohort reports in differentiated thyroid carcinoma. Within the iPTC cohort, risk factors identified in the overall analysis (male sex, larger isthmus tumour diameter, and calcification) were similarly enriched amongst patients with occult CLNM, underscoring that our nomogram primarily stratifies the probability of both clinical and occult nodal involvement rather than demonstrating an outcome benefit from therapeutic or prophylactic central neck dissection.

### Independent predictors of CLNM in simple isthmic PTC

In patients with simple iPTC, univariate analysis identified several clinicopathological and sonographic parameters significantly associated with central lymph node metastasis (CLNM): male sex (P = 0.036), greater isthmus tumour diameter (P < 0.001), presence of suspicious calcification (P < 0.001), and number of central lymph nodes cleared during surgery (P = 0.002). Details are provided in **Supplementary Table S2**.

As shown in **Table 5**, multivariate logistic regression confirmed that male sex, increased isthmus tumour diameter, and the presence of suspicious calcification were independent risk factors for CLNM, consistent with published nomograms and validation studies in comparable isthmus PTC populations. Receiver operating characteristic (ROC) analysis of tumour diameter (**Figure 4**) yielded an area under the curve (AUC) of 0.681 (95% CI: 0.594–0.769, P < 0.001), establishing an optimal cut-off of 0.995 cm. Lesions exceeding this threshold demonstrated a notably higher risk for CLNM, with specificity of 89.2% and sensitivity of 38.2%. These findings substantiate tumour size, calcific features, and sex as robust, independently significant predictors that can inform stratified surgical planning and postoperative surveillance in simple iPTC.

**Table 5:** Multifactorial logistic regression analysis of clinicopathological characteristics predicting CLNM in simple iPTC (n = 142) — Preoperative variables only (sex, tumour diameter, calcification).

Clinicopathological features	B	OR	95% CI	P-value
Gender (Male)	1.022	2.78	1.15 – 6.67	0.023
Tumour diameter (cm)	1.092	2.98	1.27 – 7.04	0.012
Suspicious calcification	1.114	3.04	1.48 – 6.50	0.003

**Figure 4:**
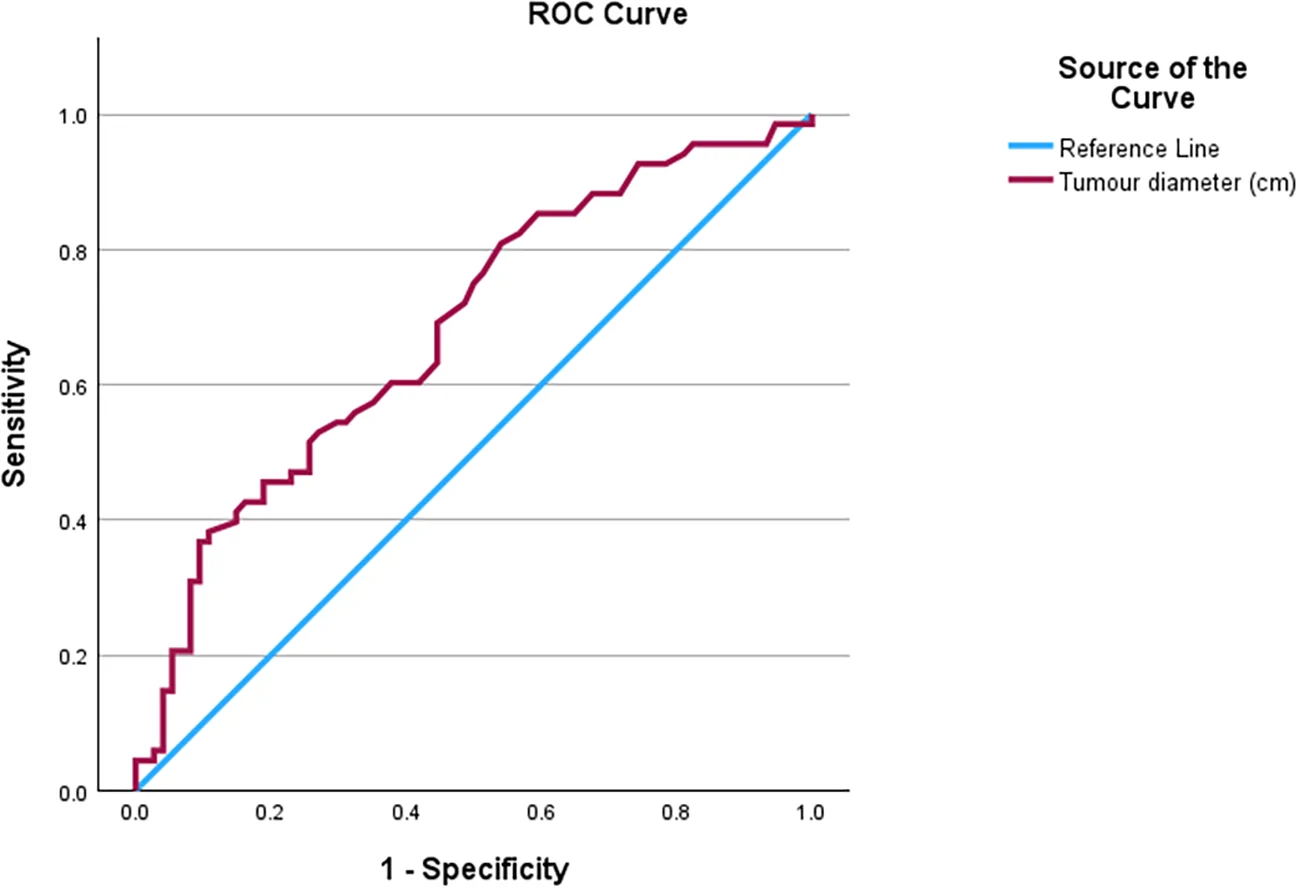
ROC curve for predicting CLNM by isthmus tumour diameter alone in simple iPTC (n = 142). AUC = 0.681 (95% CI: 0.594–0.769, P < 0.001). Optimal cutoff = 0.995 cm (specificity 89.2%, sensitivity 38.2%).

### CLNM metastatic burden, surgical extent, and postoperative outcomes in simple iPTC

Subgroup analysis showed that patients with ≥5 central lymph node metastases (CLNMs) were significantly younger (P < 0.001), with a higher proportion of individuals under 45 years (P = 0.039) and larger isthmic tumour diameter (P = 0.009). No other clinicopathological or ultrasonographic variables reached statistical significance in this high-burden group. Detailed comparisons are provided in **Supplementary Table S3**.

Comparison of operative strategies—radical isthmus resection, isthmus plus lobectomy, and total thyroidectomy—revealed that total thyroidectomy was associated with greater operative complexity and perioperative morbidity (**Table 6**). However, surgical groups differed in their baseline clinical context, and the analysis of postoperative outcomes was exploratory. Total thyroidectomy was associated with a longer operative duration and a higher frequency of postoperative hypocalcaemia; however, given the retrospective, non-randomised design, unequal group sizes, and potential confounding by indication, these associations cannot be attributed solely to the extent of surgery. No recurrence was observed during the available follow-up; however, follow-up differed significantly between groups and was insufficient to allow comparison of oncological outcomes. Additional comparative data between total thyroidectomy and non-total thyroidectomy, as well as predictors of surgical extent, are provided in **Supplementary Tables S4**, **S5**. .

**Table 6:** Comparative analysis of postoperative outcomes between surgical approaches in simple iPTC.

Clinicopathological features	A (n=10)	B (n=87)	C (n=45)	Statistic	P-value
Gender				2.368	0.306
Male	4	19	8		
Female	6	68	37		
Tumour diameter (cm)	0.72 (0.61, 0.97)	0.68 (0.46, 0.99)	0.66 (0.54, 1.15)	0.994	0.624
Calcification				0.993	0.609
Yes	7	47	24		
No	3	40	21		
CLNM ≥5				2.285	0.319
Yes	1	2	3		
No	9	85	42		
Length of surgery (min)	92.5 (80, 146)	90 (75, 115)	120 (90, 147)#	14.107	0.001
Days with tube	2 (2, 2)	2 (1, 2)	2 (1, 2)	3.058	0.217
24h postoperative drainage (mL)	36 (25, 51)	30 (23, 45)	40 (28, 55)#	7.643	0.022
Preoperative blood calcium (mmol/L)	2.42 ± 0.03	2.40 ± 0.01	2.39 ± 0.01	0.579	0.562
Postoperative blood calcium (mmol/L)	2.26 ± 0.03	2.21 ± 0.01	2.15 ± 0.02*#	5.966	0.003
Change in blood calcium (mmol/L)	0.16 ± 0.04	0.19 ± 0.01	0.23 ± 0.02	2.660	0.074
Hypoparathyroidism				19.992	<0.001
Yes	0	10	19*#		
No	10	77	26		
Follow-up time (months)	13 (10, 17)	22 (12, 29)	24 (21, 29)*	–	–
Recurrence				–	–
Yes	0	0	0		
No	10	87	45		

Group A: isthmusectomy; Group B: isthmusectomy + lobectomy; Group C: total thyroidectomy. *P < 0.05 vs Group A; #P < 0.05 vs Group B.

### Predictive nomogram for CLNM in simple isthmic PTC

As shown in **Table 5**, a multifactorial risk prediction model for central lymph node metastasis (CLNM) was developed using multivariate logistic regression, limited to independent variables with univariate P < 0.05. Each risk factor was assigned a weight proportional to its regression coefficient, allowing calculation of an individual risk score as follows: Risk Score = Σ (regression coefficient × independent variable value). This quantitative method enabled classification of patients into low-, moderate-, or high-risk groups, using the 33rd (1.5883) and 66th (2.4974) percentiles of the risk score distribution as cut-off points (**Figure 5**).

**Figure 5:**
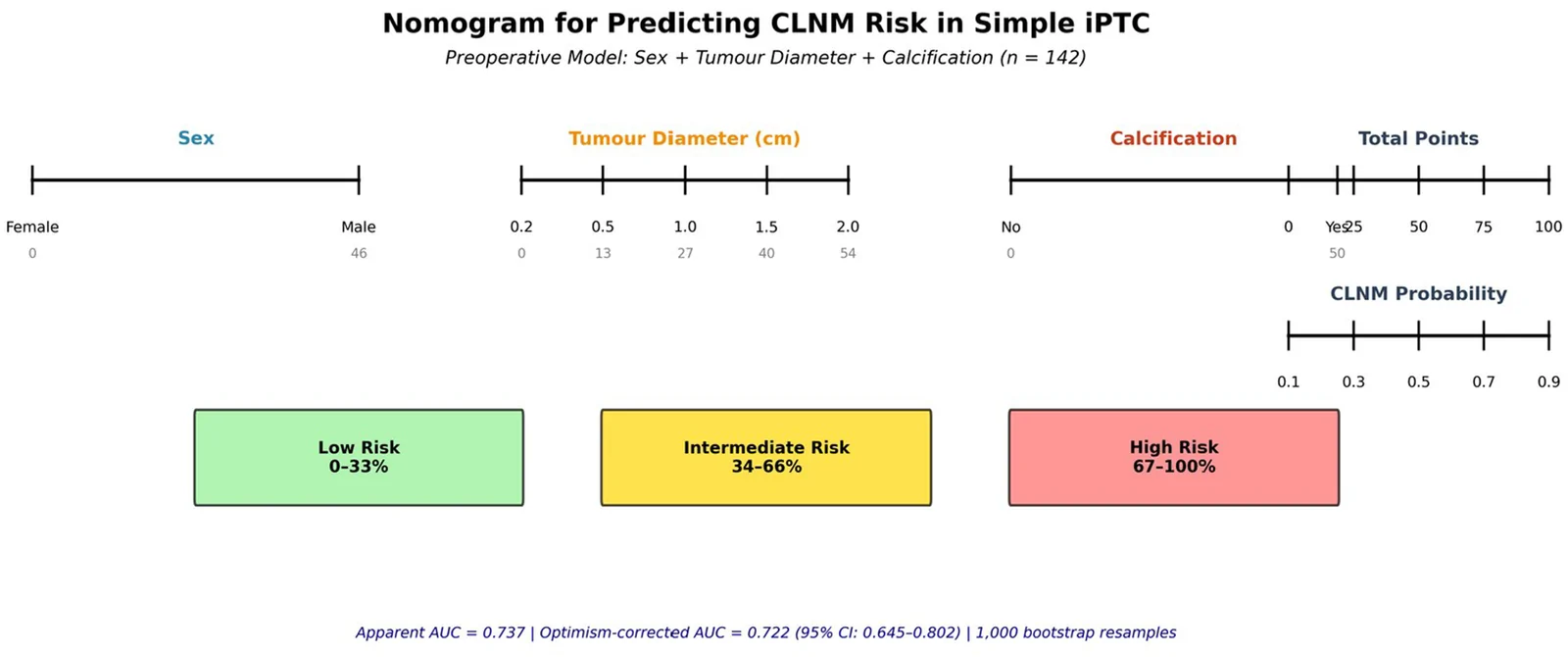
Nomogram for predicting central lymph node metastasis risk in simple iPTC based on preoperative variables (sex, tumour diameter, and suspicious calcification).

Model discrimination was assessed with an apparent AUC of 0.737 (**Figure 6**). After bootstrap internal validation with 1,000 resamples, the optimism-corrected AUC was 0.722 (95% CI 0.645–0.802), indicating acceptable discrimination for a preoperative prediction tool. Model calibration, evaluated by the bootstrap-corrected calibration slope = 0.912 and Hosmer–Lemeshow test (chi-square = 4.972, P = 0.761), showed satisfactory agreement between predicted and observed outcomes.

**Figure 6:**
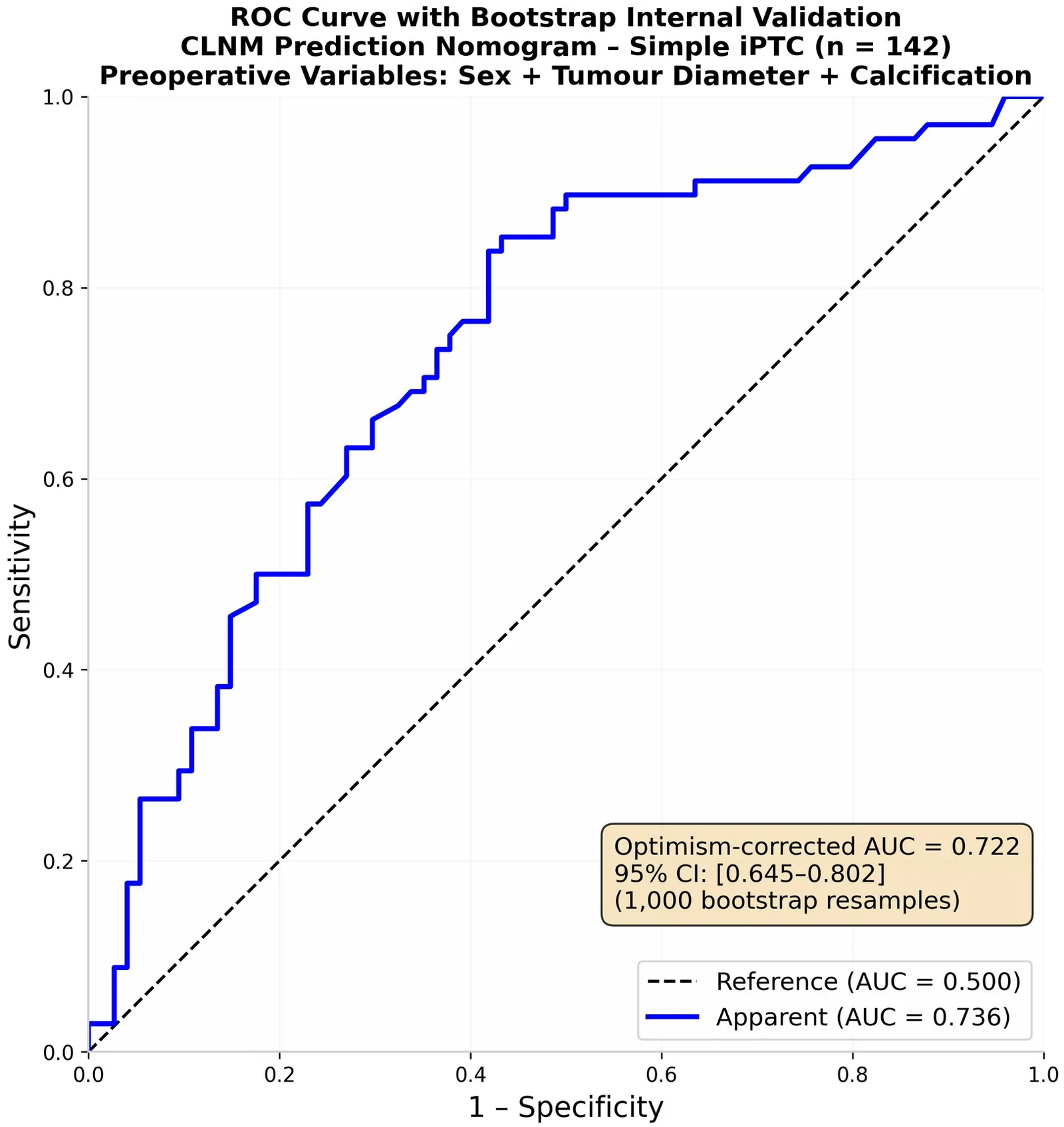
ROC curve for the CLNM prediction nomogram. Apparent AUC = 0.737; optimism-corrected AUC = 0.722 (95% CI: 0.645–0.802) after 1,000 bootstrap resamples.

This evidence-based nomogram is a practical, reliable decision-support tool for individualised surgical planning and risk stratification in simple iPTC, and aligns with recent literature validating similar predictive models for central neck metastasis in papillary thyroid carcinoma. , .

## Discussion

This study offers a comprehensive, contemporary analysis of the clinicopathological features, metastatic risk, and surgical management of iPTC, using the largest single-centre series with rigorous propensity score matching (20) and robust statistical modelling to address key clinical uncertainties in this “special location” cancer.

An important point, emphasised by current guideline recommendations and previous outcome studies, is that the presence and prophylactic removal of occult central lymph node metastases in low-risk differentiated thyroid carcinoma have not consistently led to improved disease-specific survival or durable reduction in recurrence. Our series, in which central compartment dissection was routinely performed and follow-up is of intermediate duration, is not designed to demonstrate a survival or long-term recurrence advantage of prophylactic CLND in iPTC. Instead, the data should be interpreted as providing refined risk estimates for the presence and volume of CLNM and a detailed comparison of perioperative morbidity across different extents of thyroid resection. The clinical value of this work lies in informing risk-adapted counseling and surgical planning, not in demonstrating that removal of occult nodes alone provides a survival benefit.

The data confirm and extend previous findings that iPTC displays distinct biological behaviour compared to lobe-confined PTC, with a substantially higher burden and incidence of CLNM—nearly 50% in this cohort, with an increased proportion of multifocal nodal metastasis and capsular invasion. Our propensity score–matched comparison confirms that isthmus location acts as an independent anatomic risk factor for central lymph node metastasis, even when tumour size and multifocality are rigorously balanced between cohorts. These results support the hypothesis that the unique anatomical and lymphatic environment of the thyroid isthmus—proximity to the capsule, greater lymphatic network, and potential for direct tracheal invasion—accounts for both the aggressive local behaviour and propensity for CLNM in iPTC, even when tumours are subcentimetre and in the absence of other classic high-risk features (11, 12).

This analysis advances the literature by applying a multifactorial, stratified approach to both the whole cohort and simple iPTC subgroups and provides an internally validated predictive nomogram (requires external validation) incorporating independent risk factors (tumour diameter, male sex, calcification) for CLNM. The nomogram derived from this cohort translates complex multivariable risk into a simple bedside tool that can support preoperative counseling, inform the choice between isthmusectomy, lobectomy, and total thyroidectomy, and standardize decision-making across surgeons. Notably, the cut-off of 0.995 cm for isthmus tumour diameter identified a sizeable subset of patients at high risk of nodal metastasis, whilst the nomogram’s apparent AUC (0.737) and optimism-corrected AUC (0.722, 95% CI 0.645–0.802) support its discriminatory power and clinical utility. These findings affirm—and quantify—prior observations suggesting that simple size thresholds and classic sonographic criteria alone do not adequately stratify risk in iPTC; rather, multifactorial modelling and individualised assessment are essential. Importantly, the model predicts the probability of central nodal involvement rather than survival or recurrence and should therefore be combined with existing evidence on the limited oncologic impact of prophylactic CLND when individualising surgical strategy. By stratifying patients into distinct CLNM risk categories, our model offers an opportunity to move from a “one size fits all” approach to truly individualised central neck management in isthmus PTC, whilst recognising that definitive survival benefit must be addressed in future prospective studies.

In the era of guideline-driven de-escalation, our data complement contemporary recommendations by showing that risk-adapted surgery in isthmus PTC can be safely pursued when nodal risk is quantified, rather than inferred solely from tumour size. Importantly, our outcomes data suggest that more extensive surgery (total thyroidectomy) is associated with higher perioperative morbidity—including hypocalcaemia and longer operative time—without a demonstrable reduction in short to medium-term recurrence for well-selected, favourable-risk simple iPTC. From a surgical standpoint, the absence of early nodal or distant recurrence in carefully selected simple iPTC patients treated with less than total thyroidectomy, despite a high baseline CLNM rate, suggests that maximal resection is not synonymous with superior oncologic control. The higher rate of hypocalcaemia and longer operative time observed after total thyroidectomy, without measurable short-term oncologic benefit, reinforces the rationale for limited resection plus central compartment dissection in strictly selected isthmus-confined tumours. These results are consistent with recent guideline revisions and meta-analyses advocating a risk-tailored surgical approach (including isthmusectomy or unilateral lobectomy with central compartment dissection in properly selected patients), thus supporting less-than-total resections in cases meeting strict selection criteria (tumour <1 cm, absence of gross capsule violation or preoperative nodal disease) (11, 12). Rather than using tumour diameter alone as a binary threshold for total thyroidectomy, our results highlight the need to integrate sex, isthmus tumour size, and calcification into a structured risk model when tailoring the extent of thyroid and central neck surgery.

The predominance of specific risk factors—male sex, tumour size >0.995 cm, and calcification—for CLNM has substantial clinical implications, enabling more targeted preoperative counselling, surgical planning, and postoperative surveillance. Our study also reinforces the notion that younger age is associated with a higher nodal metastatic burden, echoing population-level registry analyses (13–16) and prior reports on isthmus-specific metastatic patterns (17–19).

Supplementary analyses further demonstrated that BMI and tumour diameter were independently associated with the likelihood of total thyroidectomy, whilst the extent of nodal disease itself did not significantly drive the surgical decision (**Supplementary Tables S4**, **S5**). These findings suggest that, in this series, surgical strategy may have been influenced more by patient-related factors and tumour size than by the burden of nodal metastasis, highlighting the need for standardised, evidence-based criteria to guide the extent of thyroid resection in iPTC.

### Strengths and limitations

Strengths of this study include the large, single-centre cohort, uniform imaging protocols, strict pathological confirmation, and comprehensive prospective data collection with longitudinal follow-up. Our application of rigorous PSM minimised selection bias and enabled direct, covariate-adjusted comparison between anatomical subtypes.

The construction of a multivariable risk nomogram with bootstrap internal validation provides translational value for both clinical and research applications, although we acknowledge that external validation in independent cohorts is required before clinical deployment.

Limitations should be clearly acknowledged. In addition to its retrospective, single-centre design, this study has limitations regarding oncologic endpoints. The retrospective design, whilst mitigated by prospective data collection and blinded review, may introduce residual biases. The systematic use of central compartment dissection in this cohort precludes a direct comparison between prophylactic and no prophylactic CLND, and the intermediate follow-up duration does not allow robust conclusions about disease-specific survival or late structural recurrence. Therefore, whilst our findings refine risk stratification for CLNM and quantify the morbidity of more extensive thyroid resection, they cannot be considered evidence that prophylactic removal of occult central lymph node metastases improves long-term outcomes.

The follow-up period, though sufficient for intermediate outcome analysis, precludes definitive assessment of long-term recurrence and survival; rare adverse events, including distant metastasis and structural recurrence, require further validation in larger and multicentre cohorts.

Prospective, ideally multicentre trials with stratification by cN status and surgical strategy are required to address this question. Additionally, whilst the nomogram performed well in our population, external validation in other ethnic and surgical settings is required for full generalisability. Certain variables—such as molecular markers, details of capsular invasion, and patient-reported outcomes—were not available for all cases and warrant dedicated future investigation. Further work should also assess patient-reported outcomes and quality of life, to determine whether avoiding unnecessary total thyroidectomy in low-risk isthmus PTC translates into meaningful functional and psychosocial benefit.

## Conclusion

This study demonstrates that isthmus papillary thyroid carcinoma (iPTC) carries a higher risk of central lymph node metastasis and local aggressiveness than lobe-confined PTC, as confirmed by comprehensive clinicopathological analysis and matched patient comparison. Our results support the adoption of individualised, risk-adapted surgical management guided by key predictors such as tumour size, male sex, and calcification, with the primary goal of accurately staging central nodal disease whilst minimising unnecessary morbidity.

The internally validated nomogram developed provides a valuable preliminary clinical tool for precise risk stratification and shared decision-making regarding the extent of thyroid and central neck surgery, with optimism-corrected discrimination of 0.722 (95% CI 0.645–0.802) following bootstrap validation. Notably, within the constraints of intermediate follow-up and universal central dissection, increased surgical extent did not confer an observable reduction in early recurrence for well-selected simple iPTC, and the potential survival impact of prophylactic CLND remains to be clarified in future studies.

Limitations include the retrospective design and single-centre data. These findings lay a foundation for improved patient selection and customised intervention, and underscore the need for prospective, multicentre validation of risk models for isthmus PTC.

## Data Availability

The raw data supporting the conclusions of this article will be made available by the authors, without undue reservation.
