# Supplementary material for: Anatomical location matters: risk modelling and surgical outcomes in central lymph node metastasis of isthmus PTC
Source: Front Endocrinol (Lausanne). 2026 Sep 28;17:1922413. doi: 10.3389/fendo.2026.1922413 (PMC13654788; doi:10.3389/fendo.2026.1922413)
Supplement: Supplementary file 1 [file SupplementaryFile1.docx]

**Supplementary Material**

**Anatomical Location Matters: Risk Modelling and Surgical Outcomes in Central Lymph Node Metastasis of Isthmus PTC**

The tables compare clinicopathological features between patients undergoing total thyroidectomy and those undergoing non-total thyroidectomy (lobectomy or isthmusectomy), and identify independent predictors of surgical extent in a cohort of papillary thyroid carcinoma (PTC) patients. In univariate analysis, most demographic and tumour-related variables (including age, sex, BMI, tumour diameter, calcification, aspect ratio, extrathyroidal extension, and number of cancer foci) did not differ significantly between the two surgical groups, suggesting that the choice of procedure was not determined by these classic risk factors alone. However, multivariate logistic regression showed that BMI ≥25 kg/m² (overweight or obese per Asian criteria) and larger tumour diameter were independently associated with a lower likelihood of total thyroidectomy, while the number of central lymph node metastases and the total number of central lymph nodes removed had no significant independent effect on the surgical decision in this model. These findings suggest that, in this series, surgical strategy may be influenced more by patient-related factors (such as BMI) and tumour size than by the extent of nodal disease, highlighting the need for standardised, evidence-based criteria to guide the extent of thyroid resection in PTC.

**Contents**

1. Supplementary Tables .......................................................... 2

Supplementary Table S1. Clinicopathological characteristics of 142 patients with simple iPTC

Supplementary Table S2. Univariate analysis of clinical and pathological features in simple iPTC CLNM (n = 142)

Supplementary Table S3. Comparative analysis of clinical and pathological features between patients with simple iPTC and those with 5 or more lymph node metastases

Supplementary Table S4. Comparative analysis of clinical and pathological features between total thyroidectomy and non-total thyroidectomy

Supplementary Table S5. Multifactorial logistic regression analysis of clinicopathological characteristics affecting the surgical procedure performed

2. Supplementary Figures ......................................................... 8

Supplementary Figure S1. ROC curve of the number of iPTC lymph nodes dissected predicting the surgical procedure performed

Supplementary Figure S2. Love plot for propensity score matching covariate balance

Supplementary Figure S3. Calibration plot for the CLNM prediction nomogram

**1. Supplementary Tables**

**Supplementary Table S1. Clinicopathological characteristics of 142 patients with simple iPTC.**

| **Clinicopathological features** | **Number (%) n=142** | **Mean ± SD / Median (IQR)** |
| --- | --- | --- |
| Gender |  |  |
| Male | 31 (21.8) |  |
| Female | 111 (78.2) |  |
| Age (years) |  | 42.8 ± 12.1 |
| ≥45 | 60 (42.3) |  |
| ≥55 | 27 (19.0) |  |
| BMI (kg/m²) |  | 24.4 (22.5, 26.8) |
| <25 | 61 (43.0) |  |
| ≥25 | 81 (57.0) |  |
| Hashimoto's thyroiditis |  |  |
| Yes | 31 (21.8) |  |
| No | 111 (78.2) |  |
| Isthmus tumour diameter (cm) |  | 0.68 (0.51, 0.99) |
| <1 | 108 (76.1) |  |
| ≥1 | 34 (23.9) |  |
| Calcification |  |  |
| Yes | 78 (54.9) |  |
| No | 64 (45.1) |  |
| Aspect ratio >1 |  |  |
| Yes | 86 (60.6) |  |
| No | 56 (39.4) |  |
| Unclear borders |  |  |
| Yes | 129 (90.8) |  |
| No | 13 (9.2) |  |
| Surgery |  |  |
| Isthmusectomy | 10 (7.0) |  |
| Isthmus + unilateral lobectomy | 87 (61.3) |  |
| Total thyroidectomy | 45 (31.7) |  |
| Duration of surgery (min) |  | 100 (75, 130) |
| Days with tube |  | 2 (1, 2) |
| 24h postoperative drainage (mL) |  | 34 (25, 50) |
| Hypocalcaemia |  |  |
| Yes | 29 (20.4) |  |
| No | 113 (79.6) |  |
| Reduced blood calcium (mmol/L) |  | 0.20 ± 0.12 |
| CLNM |  |  |
| Yes | 68 (47.9) |  |
| No | 74 (52.1) |  |
| CLNM ≥5 |  |  |
| Yes | 6 (4.2) |  |
| No | 136 (95.8) |  |
| Invasion of the thyroid capsule |  |  |
| Yes | 23 (16.2) |  |
| No | 119 (83.8) |  |
| TNM stage |  |  |
| I | 133 (93.7) |  |
| II | 9 (6.3) |  |

**Supplementary Table S2. Univariate analysis of clinical and pathological features in simple iPTC CLNM (n = 142).**

| **Clinicopathological features** | **CLNM+ (n=68)** | **CLNM- (n=74)** | **Statistic** | **P-value** |
| --- | --- | --- | --- | --- |
| Gender |  |  | 4.394 | 0.036 |
| Male | 20 | 11 |  |  |
| Female | 48 | 63 |  |  |
| Age (years) | 42.47 ± 12.76 | 43.03 ± 11.60 | 0.272 | 0.786 |
| <45 | 40 | 42 | 0.062 | 0.803 |
| ≥45 | 28 | 32 |  |  |
| <55 | 56 | 59 | 0.158 | 0.691 |
| ≥55 | 12 | 15 |  |  |
| BMI (kg/m²) | 24.4 (21.9, 26.5) | 24.3 (22.6, 27.2) | -0.839 | 0.401 |
| <25 | 31 | 30 | 0.368 | 0.544 |
| ≥25 | 37 | 44 |  |  |
| Hashimoto's thyroiditis |  |  | 1.339 | 0.247 |
| Yes | 12 | 19 |  |  |
| No | 56 | 55 |  |  |
| Tumour diameter (cm) | 0.78 (0.60, 1.17) | 0.60 (0.45, 0.82) | -3.729 | <0.001 |
| Calcification |  |  | 12.923 | <0.001 |
| Yes | 48 | 30 |  |  |
| No | 20 | 44 |  |  |
| Aspect ratio >1 |  |  | 3.174 | 0.075 |
| Yes | 36 | 50 |  |  |
| No | 32 | 24 |  |  |
| Unclear borders |  |  | 0.204 | 0.652 |
| Yes | 61 | 68 |  |  |
| No | 7 | 6 |  |  |
| Number of CLN removed | 6 (5, 8) | 4 (2, 7) | -3.085 | 0.002 |
| Invasion of the thyroid capsule |  |  | 3.303 | 0.069 |
| Yes | 15 | 8 |  |  |
| No | 53 | 66 |  |  |

**Supplementary Table S3. Comparative analysis of clinical and pathological features between patients with simple iPTC and those with 5 or more lymph node metastases (CLNM).**

| **Clinicopathological features** | **CLNM≥5 (n=6)** | **CLNM<5 (n=136)** | **Statistic** | **P-value** |
| --- | --- | --- | --- | --- |
| Gender |  |  | - | 0.339 |
| Male | 0 | 31 |  |  |
| Female | 6 | 105 |  |  |
| Age (years) | 43.59 ± 11.70 | 24.00 ± 3.90 | 10.412 | <0.001 |
| <45 | 6 | 76 | - | 0.039 |
| ≥45 | 0 | 60 |  |  |
| BMI (kg/m²) |  |  |  |  |
| <25 | 5 | 56 | - | 0.084 |
| ≥25 | 1 | 80 |  |  |
| Hashimoto's thyroiditis |  |  | - | 1.000 |
| Yes | 1 | 30 |  |  |
| No | 5 | 106 |  |  |
| Tumour diameter (cm) | 0.67 (0.50, 0.98) | 1.26 (0.89, 2.91) | -2.612 | 0.009 |
| Calcification |  |  | - | 0.223 |
| Yes | 5 | 73 |  |  |
| No | 1 | 63 |  |  |
| Aspect ratio >1 |  |  | - | 0.680 |
| Yes | 3 | 83 |  |  |
| No | 3 | 53 |  |  |
| Unclear borders |  |  | - | 0.094 |
| Yes | 4 | 125 |  |  |
| No | 2 | 11 |  |  |
| Invasion of the thyroid capsule |  |  | - | 1.000 |
| Yes | 1 | 22 |  |  |
| No | 5 | 114 |  |  |

**Supplementary Table S4. Comparative analysis of clinical and pathological features between total thyroidectomy and non-total thyroidectomy in simple iPTC.**

| **Clinicopathological features** | **Total thyroidectomy (n=45)** | **Lobectomy and Isthmusectomy (n=97)** | **Statistic** | **P-value** |
| --- | --- | --- | --- | --- |
| Gender |  |  | 0.634 | 0.426 |
| Male | 8 | 23 |  |  |
| Female | 37 | 74 |  |  |
| Age (years) | 41 (19, 63) | 42 (20, 71) | -0.196 | 0.845 |
| <45 | 27 | 55 | 0.137 | 0.711 |
| ≥45 | 18 | 42 |  |  |
| <55 | 38 | 77 | 0.512 | 0.474 |
| ≥55 | 7 | 20 |  |  |
| BMI (kg/m²) | 24.8 (18.9, 37.5) | 24.2 (17.4, 40.1) | 1912 | 0.236 |
| <25 | 34 | 79 | 0.656 | 0.418 |
| ≥25 | 11 | 18 |  |  |
| Hashimoto's thyroiditis |  |  | 0.129 | 0.719 |
| Yes | 36 | 75 |  |  |
| No | 9 | 22 |  |  |
| Tumour diameter (cm) | 0.66 (0.35, 4.87) | 0.69 (0.20, 2.26) | 2070 | 0.622 |
| Calcification |  |  | 0.068 | 0.795 |
| Yes | 21 | 43 |  |  |
| No | 24 | 54 |  |  |
| Aspect ratio >1 |  |  | 0.415 | 0.519 |
| Yes | 16 | 40 |  |  |
| No | 29 | 57 |  |  |
| Unclear borders |  |  | 0.150 | 0.698 |
| Yes | 42 | 87 |  |  |
| No | 3 | 10 |  |  |
| Number of CLN removed | 6 (1, 19) | 5 (0, 16) | 1.723 | 0.043 |
| Invasion of the thyroid capsule |  |  | 0.702 | 0.402 |
| Yes | 36 | 83 |  |  |
| No | 9 | 14 |  |  |
| Number of cancer foci | 1 (1, 4) | 1 (0, 3) | 1908.5 | 0.095 |
| CLNM | 0 (0, 15) | 0 (0, 6) | 2067 | 0.582 |
| CLNM ≥5 |  |  | 0.288 | 0.591 |
| Yes | 3 | 3 |  |  |
| No | 42 | 94 |  |  |

**Supplementary Table S5. Multifactorial logistic regression analysis of clinicopathological characteristics affecting the surgical procedure performed in simple iPTC.**

| **Clinicopathological features** | **B** | **OR** | **95% CI** | **P-value** |
| --- | --- | --- | --- | --- |
| Overweight (BMI ≥25 kg/m²) | -0.948 | 0.387 | 0.171 – 0.876 | 0.023 |
| Tumour diameter (cm) | -0.767 | 0.464 | 0.220 – 0.981 | 0.044 |
| Number of cancer foci | -0.416 | 0.659 | 0.352 – 1.236 | 0.194 |
| Number of central lymph node metastases | -0.053 | 0.949 | 0.786 – 1.145 | 0.582 |
| Number of CLN removed | -0.101 | 0.904 | 0.816 – 1.002 | 0.054 |

**2. Supplementary Figures**

**Supplementary Figure S1. ROC curve of the number of iPTC lymph nodes dissected predicting the surgical procedure performed.**

**Supplementary Figure S2. Love plot showing standardized mean differences (SMDs) for covariates before and after propensity score matching (n = 268 matched pairs). Dashed lines indicate the acceptable balance threshold (|SMD| < 0.10).**

**Supplementary Figure S3. Calibration plot for the CLNM prediction nomogram in simple iPTC. The solid line represents the observed calibration curve; the dashed line represents the ideal 45° reference. The Hosmer–Lemeshow test yielded chi-square = 4.972, P = 0.761, indicating satisfactory calibration.**
